# Impact of seasonal cycle on rheumatoid arthritis based on genetic and epigenetic mechanisms

**DOI:** 10.3389/fimmu.2025.1601767

**Published:** 2025-09-05

**Authors:** Krishna Priya E.K, Shidhi P.R, Sreedevi S, Moinak Banerjee

**Affiliations:** ^1^ Human Molecular Genetics Laboratory, Division of Neurobiology, BRIC-Rajiv Gandhi Centre for Biotechnology, Thiruvananthapuram, India; ^2^ Department of Zoology, University of Kerala, Thiruvananthapuram, Kerala, India; ^3^ Department of Rheumatology, Government Medical College, Thiruvananthapuram, India

**Keywords:** rheumatoid arthritis, seasonal cycles, GWAS, genetics, epigenetics, miRNA, methylation, SNPs

## Abstract

Rheumatoid arthritis is an autoimmune disease characterized by chronic inflammation and progressive joint destruction. Genetic and environmental risk factors contribute to the development of rheumatoid arthritis (RA). Among environmental factors, seasonal cycles are reported to impact the clinical phenotypes in RA, with rheumatic flares being aggravated during the winter season. Gaining insight into the seasonal effects on molecular drivers can be vital in resolving seasonal cycles in RA pathogenesis. To understand this phenomenon, genome-wide association study (GWAS) and candidate genes were reviewed to identify their role in susceptibility to RA. Subsequently, it was verified how many of these genes are modulated by seasonal influences. Furthermore, the role of epigenetic modifications, such as DNA methylation and non-coding RNAs, involved in RA pathogenesis and whether these epigenetic effects are also impacted by seasonal fluctuation were examined. Finally, it was investigated how these genetic and epigenetic mechanisms result in the breakdown of self-tolerance and the initiation of an autoimmune action. Upon overlapping the genetic and epigenetic observations influenced by seasonal cycles, it was evident that seasonal cycles do impact the genetic and epigenetic machinery, which possibly can explain the reasons for seasonal flares in RA pathogenesis. The evidence indicates that genetic and epigenetic mechanisms are driven by climatic variation and that the timing and duration of the aberrant expression of immune response genes will drive the autoantibodies to develop seasonal flares of RA.

## Introduction

Rheumatoid arthritis (RA) is a chronic autoinflammatory joint disease characterized by synovial hyperplasia, loss of self-tolerance, and progressive bone erosions. The prevalence rate of RA is 1% in the global population (1). Autoantibodies are the major hallmark of RA, and the presence of autoantibodies like RF and anti-cyclic citrullinated peptide (anti-CCP) antibodies (2) in strong positivity has a significant impact on the etiopathogenesis of RA. These autoantibodies are very specific for RA, are present in the earlier stages of the disease, and also correlate with the severity of the disease (3). In rheumatoid arthritis patients, both RF and anti-CCP levels vary in each individual and exhibit high diversity in the various global populations (4). The variability in the autoantibody status may be due to gene–environment interaction in various geographical landscapes, which strongly impacts the increased expression of serological markers like RF and anti-CCP in RA.

The genetic architecture and environmental factors underlie the crucial mechanism in RA pathogenesis (5). Evidence related to clinical phenotype and genealogical data suggests that occurrences of RA are greater in the family pedigree of the proband [6] and in twins, with 50% genetic heritability in monozygotic twins, which display a better concordance rate than dizygotic twins, thereby suggesting a strong contribution by the genetic elements in RA pathogenesis (7). The nature of environmental perturbation in the disease outcome is ambiguous. While environmental factors like smoking and hormonal factors are known to influence the development of RA, the significance of factors such as day and night length, temperature, humidity, seasonal variations, and global warming cannot be ruled out, as these factors can also impact gene expression and epigenetic influence on immune response genes. The incidence and impact of smoking are also dependent on seasonal influences. Seasonal rheumatic symptoms are commonly reported across all rheumatic diseases with bimodal patterns of seasonality for global severity, joint pain, fatigue, and socialization (8). Seasonality defines the variations in the seasons, like winter, summer, spring, and autumn. The genetic determinants and environmental factors are intertwined with lifestyle in determining the health status of an individual, where gene–environment interaction strongly influences epigenetic mechanisms like DNA methylation, histone modifications, acetylation, and miRNA expression (9). Thus, epigenetic regulation defines how gene–environment interaction impacts DNA modifications without altering the genomic domains. These environmental factors can result in epigenetic disarray, thereby influencing RA flares through the ubiquitous expression of host immune system genes, resulting in the breakdown of immune tolerance and initiating autoinflammatory responses in genetically predisposed RA individuals (10). RA flares are referred to as RA symptoms that are altered during the shift in seasonal changes. This review attempts to understand how genetic and epigenetic phenomena are associated with RA and to evaluate their relationship to seasonal variations and how these seasonal factors can promote or aggravate the progression of rheumatic phenotypes. To address this, we first try to demonstrate if RA flares can be aggravated during seasonal cycles, then try to identify the genes that are associated with RA risk and how many of these genes are likely to be influenced by seasonal cycles, and subsequently assess the directional shift of different epigenetic events such as DNA methylation and miRNA expression in RA and how many of these directional shifts in epigenetic events are caused by seasonal cycles.

## Epidemiology of rheumatoid arthritis

As per the global and regional prevalence estimates, rheumatoid arthritis is predicted to increase by 80.2% (63.3%–92.1%), reaching 31.7 million people globally by 2050, with the highest burden in Andean Latin America (11, 12). Prevalence and incidence studies on rheumatoid arthritis have reported considerable global variation. An increased prevalence rate was observed in Europeans, in sharp contrast to Asians. Studies have reported a higher prevalence of RA, with 0.5%–1.1% in Northern European and North American populations (13, 14) compared to 0.3%–0.7% in South Europeans (15) and 0.2%–0.3% in Caribbean populations. Even among Asians, the incidence rate of RA was found to be higher in the Japanese population with 0.2%–0.3% (16) but lower in the Indian population (0.1%–0.3%). Studies have shown that the incidence and risk of RA are higher in regions at higher latitudes, which are typically found in the Northern Hemisphere. It has been reported that RA is more common in Northern Europe and North America than in Southern Europe, Africa, and the Southern Hemisphere (17). Potential explanations for these regional variations could be meteorological factors like climate, environmental exposures, dietary factors, behavioral factors, RA diagnosis, or genetic factors (8, 15, 18). Environmental factors like climate change, humidity, temperature, pollution, smoking, and disease-causing microbes can also cause RA (15, 19, 20). It is well known that dietary factors can also vary in different ethnic groups, which can differentially impact RA status (21). Hence, variations in these demographic factors could reflect differences in the prevalence rates of the diseases, which can further be determined by their inherent genetic background and through their impact on epigenetic phenomena, indicating that the genetic factors could mediate the threshold of environmental impact. It is the allelic background that will define the ability to withstand environmental impact and thereby define the threshold, as evident from the genes’ allele-specific expression. These geographical, environmental, climatic, and genetic factors may determine the variation in RA flares. However, how climatic conditions or seasonal variations impact the development of RA has never been the focus of a detailed investigation. This perspective article attempts to investigate genetic predisposition to RA and how genetic predisposition factors are modulated by environmental factors with specific reference to seasonal changes. Evaluating the relationships between genetic, epigenetic, and seasonal factors may help in refining the understanding of pathogenesis in autoimmune diseases with respect to seasonal RA flares.

### Genetic components of rheumatoid arthritis

Genetic polymorphisms are highly inheritable variations in DNA sequences that exhibit a significant role in the etiology of RA. Many single-nucleotide polymorphisms (SNPs) are likely to have a role in individuals’ predisposition to develop RA, which in turn can contribute to phenotypic variation, increased disease susceptibility, and altered genomic expression and function (22). Hence, identifying predisposed polymorphisms that could play a substantial role in defining immunogenetic impact on the pathogenic mechanism of RA is critical. Candidate gene studies are based on a strong hypothesis, while genome-wide association studies (GWASs) provide a powerful genome-wide approach to understanding the genetic basis of complex traits. Unravelling the role of susceptibility loci in the development of complex disease conditions like RA by assessing its contribution toward genetic heritability can be challenging, but understanding this genetic basis will help in defining the threshold of an individual to withstand environmental impact. GWAS and candidate gene observations for RA have identified factors within human leukocyte antigens (HLAs) and non-HLA genes.

### HLA genetics associated with RA and its global variation

HLAs are encoded by major histocompatibility complex (MHC) class I and II genes, which are highly accountable for the regulation of innate immune responses during pathogen invasion. Many immune cells are central to RA pathogenesis, where CD4+ effector memory T cells are specifically expressed on HLA-DR genes located within RA-associated loci in chromosome 6 (23). HLA class II encodes for DR, DQ, and DP loci (classical regions) and DM and DO loci (non-classical regions) where allelic combinations exist in the classical DR regions within the third hypervariable region (HVR3) of the HLA-DRB1 chain termed as shared epitopes (SEs), showing an increased risk for developing RA (24, 25). The shared epitope hypothesis is attributed to the presence of arthritogenic antigens (26) and T-cell repertoire selection (27). SE in the HLA-DRB1 region exhibits a remarkable role in inflammatory regulation and immune responses during RA pathogenicity (28, 29). Interestingly, shared epitope-coding *HLA-DRB1* allelic risk combinations are strongly associated with autoantibodies like anti-CCP (ACPA) in various global populations (**Table 1**) (30–45). SE-coding HLA-DRB1 alleles have been shown to be associated with the severity of disease and also exhibit allele-dose effect; i.e., patients with two SE-coding alleles tend to experience more severe disease than patients with one allele, who, in turn, have more severe RA than SE-negative patients. In a Dutch population-based inception cohort, the OR of the association of two copies of the SE allele with anti-CCP positivity was 11.79 (p < 0.0001), while the OR for just one SE allele was 4.37 (p < 0.0001). Similarly, in North American RA Consortium families, linkage and association analyses revealed that SEs are associated only with anti-CCP-positive disease and not with anti-CCP-negative disease. Anti-CCP antibodies primarily mediated the association of the SEs with joint damage or disease persistence (46). SE-positive individuals also show a lower likelihood of refractory RA, indicating a potential link between shared epitope status and treatment efficacy (47). That HLA-DRB1 13:01/13:02 is a risk factor for Koreans while showing a protective effect in the Japanese population may be due to the cross-reactivity between citrullinated proteins and the presence of certain specific sequences in DRB1, which encodes for the same autoantibodies, or HLA-DRB1 allele associated with the lower frequencies in the population may have a protective effect (48) due to the inter-individual variation in host immunogenetic background. The geographical distribution of HLA-shared epitopes and their association with anti-CCP and RF in rheumatoid arthritis development show huge differences in the global population, indicating a complex hierarchy conferred by HLA-DRB1 and its allelic combinations in Caucasians (49) and East Asian populations (48). Therefore, the allelic heterogeneity in HLA-shared epitopes in Europeans and East Asian populations suggests that HLA loci may bear the signature of selective pressure in which selected epitopes influence various expression patterns of autoantibodies in RA individuals. The role of environmental interaction with these shared epitopes also influences the expression of the genes associated with autoantibodies directly or indirectly. The increased and decreased distribution of anti-CCP and RF may be due to the influences of environmental or seasonal parameters on disease variants.

**Table 1:** HLA-DRB1 alleles associated with ACPA and RF for RA susceptibility in global populations.

HLA-DRB1 locus	Risk	P-value	OR	ACPA/RF	Populations	References
*04:01/*04:03	Risk	0.001	3.74	Increased ACPA	Europeans (French)	(25, 26)
					Latin Americans	(27)
				Increased RF	East Asians (Koreans)	(28)
*04:04/*04:08	Risk	0.001	4.2	Increased ACPA	Europeans	(29)
*04:05	Risk	0.0001	5.6	Increased ACPA	Koreans	(30, 31)
					Japanese	(32, 33)
	Risk	0.02	4.7		Singaporeans	(34)
*01:02/*01:01	Risk	0.001	1.44		Europeans (Israeli Jews)	(35)
*14:02	Risk	0.001	1.8	Decreased ACPA	Native Americans	(36)
*14	Risk	0.001	0.48	Decreased ACPA	Europeans	(28)
					African American	(37)
*10:01	Risk	0.031	1.9	Increased ACPA	East Asians (Japanese)	(38)
					Singaporeans	(34)
*07:01	Risk	0.0001	0.91	Decreased ACPA	East Asians (Koreans) (Singaporeans)	(30, 31)
*14:05	Risk	0.001	0.31	Decreased ACPA	East Asians (Japanese)	(39)
*0901	Risk	0.001	1.38	Increased ACPA	Korean	(31)
*13:01/13:02	Risk	0.001	0.31	Increased ACPA	Koreans	(31)
*15	Risk	0.001	0.69		Europeans	(40)

*ACPA, anti-cyclic citrullinated peptide antibodies; RF, rheumatoid factor; RA, rheumatoid arthritis.

### Non-HLA genetics associated with RA

Apart from HLA, numerous non-HLA risk loci susceptible to RA have also been identified. These genetic markers are tightly linked with the various functions of the gene, molecular mechanisms, and signaling pathways. Highly polymorphic regions in non-MHC genes strongly influence disease phenotypes, which involve the alteration in transcriptional activity, miRNA regulations, splicing mechanism, mRNA stability, and translational and post-translational modifications. Earlier studies have indicated that SNPs of non-HLA genes like *PTPN22*, *RAF1*, *CTLA4*, *IRF5*, *STAT4*, *FCGR3A*, *IL6ST*, *IL2RA*, *IL2RB*, *CCL21*, *CCR6*, and *CD40* are strongly associated with RA susceptibility (50).

Several SNPs of the immune regulatory genes and non-MHC genes have been identified, but their association with clinical phenotypes remains elusive. Over the years, GWASs have identified several risk loci (**Table 2**) and even candidate genes (**Table 3**), which have demonstrated the presence of plausible risk variants that are significantly associated with RA in different global populations. However, there seems to be a lack of consensus across studies to replicate these observations demonstrating significant ethnic variation (**Tables 2**, **3**). These variations in GWAS or candidate genes could reflect their role in genetic, phenotypic, or environmental heterogeneity. It could also demonstrate differential selection in immune response genes, possibly due to environmental adaptation.

**Table 2:** GWAS genes associated with Rheumatoid Arthritis in global populations.

Chromosomes	Chromosomal Position	Gene	European Populations						East Asian Populations			South Asians	African Americans
			UK	Spain	Sweden	Germany	North Ireland	Denmark	Japan	Korea	China	Indians	Africans
Chromosome 1	1p13.2	PTPN-22	**s**	**s**	**s**	**s**	**s**	**s**	**s**				
	1p36.2	PAD14	**s**		**s**		**s**		**s**				
	1p13	CD2, CD58	**s**		**s**	**s**	**s**						
	1p23	FCGR2A	**s**		**s**		**s**		**s**	**s**			**s**
	1q31	PTPRC	**s**		**s**		**s**		**s**	**s**		**s**	**s**
Chromosome 2	2p16.1	REL			**s**		**s**						
	2p14	SPRED2	**s**				**s**		**s**				
	2q32	STAT4	**s**	**s**	**s**					**s**			
	2q11.2	AFF3	**s**				**s**		**s**			**s**	
	2q33	CD28	**s**			**s**		**s**				**s**	
	2q33.2	CTLA4	**s**		**s**				**s**	**s**			
Chromosome 4	4p15	RBPJ	**s**		**s**		**s**						
Chromosome 5	5q11.2	ANKRD55, IL6-ST	**s**				**s**					**s**	
Chromosome 6	6q27	CCR6	**s**		**s**		**s**		**s**			**s**	
	6q21	TNFAIP3		**s**					**s**			**s**	
	6p21.32	HLA-DRB1	**s**		**s**		**s**		**s**	**s**			
Chromosome 7	7q32	IRF5		**s**	**s**				**s**				
Chromosome 9	9p13.3	CCL21	**s**				**s**						
	9q33.2	TRAF1-C5	**s**	**s**							**s**		
Chromosome 10	10p15	IL-2RA	**s**				**s**		**s**	**s**		**s**	**s**
Chromosome 11	11p12	TRAF6	**s**		**s**					**s**			
Chromosome 20	20q13	CD40			**s**				**s**			**s**	
Chromosome 21	21q22.3	UBASH3A	**s**		**s**		**s**		**s**	**s**			**s**
Chromosome 22	22q12	IL-2RB	**s**		**s**	**s**	**s**					**s**	

**s** stand for Significantly associated.

**Table 3:** Candidate genes associated with Rheumatoid Arthritis in global populations.

Genes	European Populations							East Asian Populations			South Asian Populations	
	UK	Spain	Sweden	Germany	Denmark	France	Switzerland	Japan	Korea	China	North Indians	South Indians
TNF		+									+	+
TNFRS1A	+									+		
TNFRS1B	+	+			+							
IL-10	+	+	+		+	+				+		+
IL-1B					+			+		+		+
IL-1A	+			+				+		+		
IL-18	+								+	+		
IL-6	+	+								+	+	+
IL-4 VNTRS		+	+			+	+				+	
CTLA-4		+		+		+		+		+	+	
CD-40	+							+	+		+	
MMP1	+			+		+				+		
MMP3	+			+		+				+		
LTA	**+**	+										

**+** stands for Positively associated.

**Figure 1:**
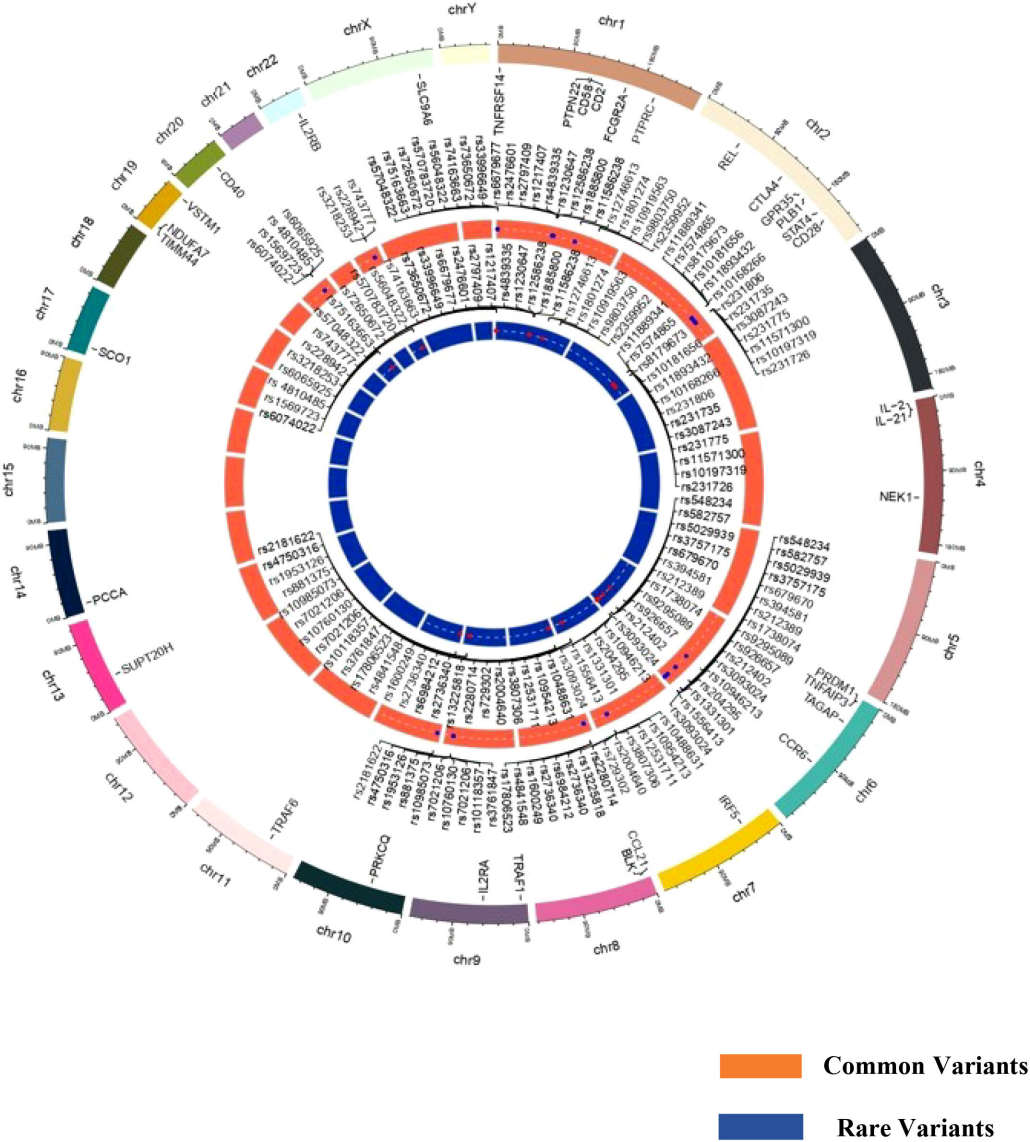
Circos plot representing common and rare variants associated with rheumatoid arthritis susceptibility.

The distribution of GWAS genes susceptible to RA based on common and rare variants is presented in **Figure 1**. The majority of the risk genes were spotted on chromosomes 1, 2, and 6 (**Supplementary Table 1**), which are also considered to be hotspots for developing RA in the global population. Several population-specific rare variants in RA-susceptible genes have been reported in European and East Asian populations (Japanese population) but have lacked replication in other populations. Evidence from whole-exome sequencing has identified several rare variants and common variants in the contribution of *PTPN22* to the risk of RA in the European population, but not in the Korean population. Interestingly, many of these risk variants lie within the coding regions of a gene, which can have a functional role in modulating disease traits (51). This could also reflect a population-specific genomic selection associated with environmental or climatic shifts, providing a selective advantage for certain alleles. The severity of the diseases and the expression of various clinical phenotypes may be largely due to the burden of rare variants in individual loci (52). The individuals carrying the risk variants may have a reduced threshold to withstand environmental risk compared to non-risk variants. Hence, understanding the role of rare variants in deriving the genotype-specific environmental threshold to withstand an adverse environment and their relationship to clinical parameters may provide insight into immune signaling pathways, especially cytokine expressions, which are dysregulated during an inflammatory condition.

### Seasonal influences in RA pathogenesis

Many autoimmune diseases are strongly influenced by climatic perturbation (53). Studies on the effect of seasonal variations on RA and its symptoms are sparse, but seasonal variations are known to impact differentially. The prevalence of RA in East Asian and Northern Europeans is higher than that in South Asian populations, showing the impact of climatic variations on the geographical landscape for the increased expression of autoantibodies for developing RA (19). Several meteorological factors, including temperature, humidity, and barometric pressure, have been linked to RA symptoms. Low temperatures and high humidity, especially during winter months, are often associated with increased pain and flare-ups in RA patients (54). Conversely, warmer, drier conditions may provide some relief. Barometric pressure changes, particularly low pressure, have also been suggested to worsen RA symptoms. Decrease in barometric pressure and increase in humidity are associated with increased risk of RA. It has been reported that if barometric pressure decreases, the hydrostatic pressure between cells increases, causing edema or swelling. The increase in hydrostatic pressure induced mRNA expressions of interleukin 6 (IL - 6), TNF-alpha (55), and interleukin 8 (IL - 8). Even mechanical stress-induced expression of IL - 8 and IL - 6 has been reported in human periodontal ligament cells. Mast cells may be influenced by barometric pressure and may be an important source of TNF-alpha and IL - 6 (56, 57). Relative humidity has been reported to influence interferon gamma (IFN-gamma), which is known to intensify inflammation. Change in weather with specific reference to barometric pressure and relative humidity can stimulate the expression of IL - 6, which in turn can influence a cascade of positive feedback loops with IL - 17, resulting in destructive arthritis (54, 57).

Studies in the Japanese population have shown that RA activity is aggravated maximally during the spring and winter seasons but is lower in autumn (58), indicating the effect of seasonal fluctuations on disease activity and increased RA flares. Moreover, RA flares worsen during the winter season than in any other season and are strongly associated with the severity of the disease (59). Extreme climatic variation impacts the pain levels, mood swings, and physical activities of RA individuals, which worsens RA symptoms (19). Seasonal changes can influence factors like IgG antibody levels, impacting the inflammatory process in RA. RA patients were more likely to report tender joints with minimal temperature increase and maximal temperature decrease in winter. The inverse effect was observed in summer; specifically, patients had more painful joints when the daily minimum temperature decreased and the maximum temperature increased (60). These observations clearly demonstrate the role of climatic effects or latitudinal effects on the altered expression of autoantibodies in developing rheumatic phenotypes, which can also be indicative of differential prevalence in geographical distribution. This differential impact of seasons and ethnicity may be influenced by their genetic makeup or differential epigenetic influence. RA is an autoimmune condition, which helped us to focus on the role of immune response genes and how they are impacted by seasonal cycles.

**Figure 2:**
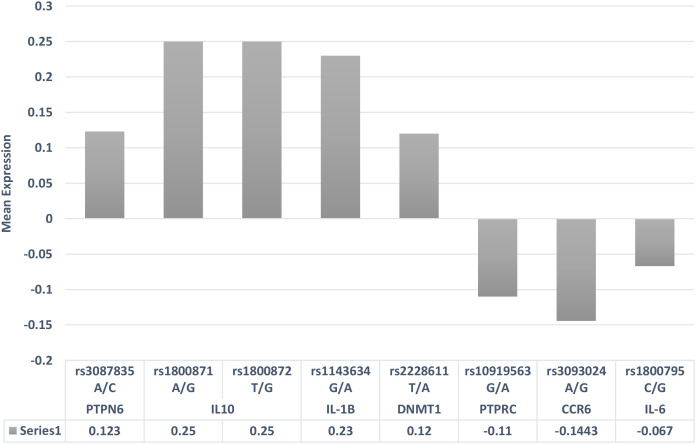
eQTL gene expression patterns of risk variants associated with rheumatoid arthritis. eQTL, expression quantitative trait locus.

Health and disease conditions display annual periodicities, but it is rarely investigated how climatic perturbations can contribute to these periodicities in maintaining health and what the molecular events are. Emerging lines of evidence have indicated that ~23% of the genome is impacted by seasonal variations, which involves several immune response genes that are differentially expressed during the winter and summer seasons (61). This upregulation or downregulation of immune response genes during different seasonal cycles indicates that they are trying to maintain immune homeostasis under different physiological conditions due to different seasonal cycles (62). When these seasonally differentially expressed immune response genes are overlapped with GWAS and candidate gene risk variants with seasonal cycles, one can possibly resolve why, in the winter season, certain biological pathways trigger the disease activity pertaining to RA pathogenesis (**Table 4**). The allelic risk variants in these genes may further define the threshold ability to maintain immune homeostasis. While assessing the GWAS genes, it was observed that the allelic expression of some of these risk variants, based on the expression quantitative trait locus (eQTL) expression extracted from the GTEx data, had differential expression patterns (**Figure 2**). Even the variation in the gene expression levels may be due to the burden of variants and the linkage disequilibrium pattern of these causal variants (63), which may cause more aggressive RA flares. The overrepresentation of the genetic risk variants of *PTPN6*, *IL-10*, *IL-1β*, and *DNMT1* genes in RA was found to be upregulated, while *PTPRC*, *CCR6*, and *IL-6* genes (**Figure 2**) were downregulated according to eQTL (https://gtexportal.org/home/datasets). In addition to shared epitopes, other genetic markers associated with RA include *STAT4*, *TRAF1/C5*, and *PTPN22*. These genes also play a role in immune system regulation and inflammation, which are key processes in RA development. The differential expression of these risk variants will define the threshold of expression for different seasons, which in turn will define the influence of seasons on immune homeostasis, resulting in RA pathogenesis and severity. Thus, seasonal variations coupled with genetic risk variants can become the primary driver for immune response genes, leading to a crosstalk as revealed by genetic and epigenetic studies.

**Table 4:** Differential expression of immune genes and their corresponding rheumatoid arthritis-associated genes in response to seasonal variations.

Seasonal variations	Immune genes upregulated	RA-associated genes	Immune genes downregulated	RA-associated genes
Winter season	PTPRC, ALDH2, PLCB2, PLCG2, PPAP2B, RKCB, PSME2, RASGRP1, LRCC1 IL - 32, IL - 2R, CL18, CXCL9, AIM2, ADAMDEC1, ALOX5, CYFIP2, EDNRB, EIF4E2, HLA-DOB, HLA-F, SDC1, FCGR3A, FCGR2A, ARNTL, MAPK8, CCL18, TRAF3, PTPN6, CYP4FP, IL - 6, IL - 1B, TNF-alpha, SHMT2, DNMT1, TLR2, SOCS3, TNFRSF10C, CXCR1, CXCR2, JAK2, FGR, CD22, NFKB1	PTPRC PLCG2 FCGR3A FCGR2A DNMT1 IL-6 IL-1B TNF-alpha CCL18 CXCL9 JAK2 ARNTL EDNRB	PTTG1, FZD2, IRF8, PSMB8, PARP1, NAGA, CCR5, TNFAIP8, IRF9, CD46, IL15, CD38, IL - 10, TNF-R1, TNF-R11	CCR6 IL-10 CCR5 PARP1 IL-15 TNF-R1
Summer season	NUP55, EIF3J, PAIP1, HSPA9, RAE1, DDX6, SLU7, NFKB1, PIK3CA, c19orf10, CUL2, SLU7, FRS2, MAPK1	MAPK1	HLA E, HLA A, FOX1, APEH	HLA-A FOX1

RA, rheumatoid arthritis.

Evidence has shown that cytokine dysregulation is associated with the development of RA (64). The expression pattern of cytokine alterations depends on the tissue types and Th1 and Th2 responses, which are known to regulate the homeostatic balance in the human immune system. The disarray in the cytokine levels can lead to tissue destruction and bone erosions in RA. In rheumatic conditions, IL - 6 pro-inflammatory cytokines predominate over anti-inflammatory cytokines in the development of chronic inflammation (65). In fibroblastic cells, the expression pattern of IL - 6 cytokine was found to be downregulated compared to that in other tissues, while the overexpression of the same cytokine was observed in circulating blood. This pleomorphic pattern of cytokine gene may be due to the disparity in allelic distribution (66) or epigenetic interaction. The upregulation and downregulation of pro-inflammatory and anti-inflammatory cytokines are completely dependent on environmental conditions, and their activity during immune responses is determined by the variants in the cytokine genes. The eQTLs of the risk variants of immune response genes and their allele-specific expression overlapped with the seasonal cycle gene expression (**Figure 2**). Interestingly, the risk alleles do indicate risk-specific thresholds for seasons, which may help in understanding the variations in RA flares across seasons. Seasonal variations can also impact the synergistic action of cytokine expression by altering the physiological nature of immune cells, which reflects the inflammatory responses in RA.

Among the HLA genes, HLA-B27 is considered to be the diagnostic feature of RA, and the complications in RA associated with uveitis have been reported to be higher with the seasonal fluctuations in November to February in the Indian population (67). Thus, seasonal variations between months of the year, which include differences in day length, UV index, humidity, and temperature, potentially influence immune response genes that are expressed independently and interactively. Evidence suggests that HLA-B27 is also related to arthritic spondylitis, where HLA was found to be highly associated with the winter season of the year (68). The SEs linked with RA, disease phenotypes, and genetic heritability can amplify the gene-dose-dependent variable, where individuals carrying one or two SE-coding alleles have increased susceptibility to RA compared to SE-negative individuals with the HLA-DRB1 gene (42). Hence, understanding the role of HLA genes with different SEs and their association with various phenotypic expressions (69, 70) and anti-CCP levels (46, 71, 72) in RA individuals can help in resolving the HLA complexity in diseased individuals. The mechanism of action of HLA and its interaction with T cells in maintaining homeostatic balance in the immune system is ambiguous. Evidence suggests that the HLA alleles do reflect seasonal cycles, with HLA-DR3-positive cases showing a seasonal diagnosis pattern for diabetes independent of age, while DR3-negative cases showed an age-dependent seasonal pattern (73). Thus, understanding the complexity behind the HLA genetics and gene–environment interaction with seasonal variables could provide a significant role of HLA SEs in RA origin. Therefore, understanding the real mechanism behind the gene expression pattern and its seasonal periodicities in RA and its severity may resolve clinical and phenotypic heterogeneity.

## Epigenetics of rheumatoid arthritis

Genetic architecture could explain the involvement of genetic variants in resolving their role in RA pathogenesis and its phenotypic variation to an extent, while RA is also reported to be extensively influenced by gene–environment interaction. An epigenetic mechanism is defined as the stable phenotypic change resulting from a chromosomal alteration without changing the DNA sequences (74). While several environmental factors are reported to influence RA pathology, the scope of the present perspective includes determining if seasonal variations can be critical to RA pathogenesis and its seasonality. Epigenetic players consist of epigenetic “writers” that modify DNA and histone proteins, epigenetic “readers” with specific protein domains that recognize DNA or histone marks, and then epigenetic “erasers” that can remove the existing signals for new modifications (75, 76). Many studies have suggested that frequent errors in epigenetic phenomena lead to the miswriting, misreading, or defective removal of epigenetic signals in a pathological condition (77). The methylation of CpG islands with their hypo- and hypermethylation, post-translational modifications to the amino-terminal of histone proteins, and long non-coding RNA (lncRNA) expression contributes to the major epigenetic events that are mediated by the effect of gene–environment interaction (78, 79). Therefore, understanding the epigenetic landscape of seasonal variations and overlaying it with the seasonality of RA flares may provide a novel insight into the molecular events associated with seasonality in RA pathogenesis.

### DNA methylation and histone modification in rheumatoid arthritis

DNA methylation is an important epigenetic modification that is involved in aberrant gene expression and transcript splicing. Methylation patterns in T cells, B cells, peripheral blood mononuclear cells (PBMCs), and synovial fibroblastic cells could provide a complete spectrum of epigenetic patterns of various immune cells in the disease pathogenesis of RA (**Table 5**) (80). Studies have suggested that in rheumatic conditions, abnormal DNA methylation has been reported in the earlier stages of the disease (81). Altered DNA methylation pattern influences the physiological and functional characteristics of immune cells, which directly or indirectly affects the overexpression of inflammatory markers like RF and anti-CCP for RA severity.

**Table 5:** Differential CpG methylated sites in T- and B-lymphocytic cells in rheumatoid arthritis.

Cell types	Gene	Chromosome	CpG sites	Methylation status
T lymphocytes	C1orf106	1	cg10092377	Hypermethylated
		2	cg20891558	Hypermethylated
		2	cg05890377	Hypermethylated
	ARSB	5	cg24166916	Hypermethylated
		6	cg23681866	Hypermethylated
		6	cg24838316	Hypermethylated
		6	cg26121931, cg15411272	Hypermethylated
	HIST1H4D	6	cg21438527	Hypermethylated
	DUSP22	6	cg03395511, cg11235426, cg15383120, cg 20619695	Hypermethylated
		6	cg26121931, cg15411272	Hypermethylated
	ZFP57	6	cg26021304	Hypermethylated
	LSM5	7	cg26856631	Hypermethylated
		16	cg04412904	Hypermethylated
T lymphocytes	TMEM9	1	cg16112880	Hypomethylated
		3	cg01412404	Hypomethylated
	PDHB	3	cg07576186	Hypomethylated
		3	cg01412404	Hypomethylated
		6	cg20228636, cg10471638, cg13835168	Hypomethylated
			cg 1019916	Hypomethylated
	DDO	6	cg11645539	Hypomethylated
	MGMT	10	cg09993319	Hypomethylated
	CCS	11	cg24851651	Hypomethylated
	FBRSL1	12	cg12093005	Hypomethylated
	GALNT9	12	cg05229598	Hypomethylated
		14	cg 21792493	Hypomethylated
Whole blood	IL-10	1	cg14180511	Hypomethylated
			cg04321197	Hypomethylated
B lymphocytes	ASB1	2	cg09969882	Hypomethylated
	PCDH12	5	cg18849725	Hypomethylated
	MSH5	6	cg23634079	Hypomethylated
	MGMT	10	cg09993319	Hypomethylated
	BARX2	11	cg22744587	Hypomethylated
	CCS	11	cg24851651	Hypomethylated
	GPR133	12	cg01008088	Hypomethylated
	AHSP	16	cg08624915	Hypomethylated
	PLEKHA4	19	cg15549821	Hypomethylated

Methylation that occurs in the various immune cells determines the host’s immune responses toward a pathogenic condition. Global DNA hypomethylation has been reported in immune cells like T cells and monocytes in RA patients (82) and could be responsible for the overexpression of inflammatory cytokines in the synovial fluid (83). Studies have suggested that pro-inflammatory cytokines could suppress DNMT gene expression in synovial fibroblasts, giving more evidence to the global hypomethylation status of key genes involved in RA pathogenesis. Methylation that occurs in the CpG sites in immune cells shows the varying effects on gene expression, where intragenic methylation is involved in the regulation of alternative splicing mechanisms, the prevention of spurious transcription, and the expression of regulatory ncRNA. Hence, it is very important to know single cell types that are predominantly involved in the epigenetic regulation of genes and interpret the threshold of cell types that trigger biologically relevant events in the immune system. Genome-wide DNA methylation by microarrays revealed that alterations in B-lymphocytic cells influence the early progression of RA without drug implementation compared to healthy controls (84). The promoter region of the GWAS gene CTLA - 4 (−658 CpG) was found to be hypermethylated in RA individuals, amplifying the aberrant function of regulatory T cells where hypermethylation prevents the binding of the nuclear factor of activated T cells (NF-AT) with a cytoplasmic one, called NF-ATc2, which leads to the decreased expression of the CTLA - 4 gene in RA patients (85). Other microarray studies have identified 10 differentially methylated CpG sites that are localized on 6p12.1 and form two separate loci containing MHC, which also confirms the relevant role of DNA methylation on these regions in determining RA (86). Thus, heterogeneity in various cell populations expressing irregular methylation patterns compared to healthy donors demonstrated that the cg23325723 CpG promoter site methylation in PBMCs was strongly associated with RA development (87). Moreover, DNA methylation is a highly stable epigenetic phenomenon, and its cell-type specificity provides an alternative path for cellular deconvolution in clinical settings (88). Methylome studies on synovial fibroblastic cells have also shown differentially methylated loci that alter the epigenetic architecture of genes such as *TRAF2*, *TIMP2*, *CCR6*, *IL-6R*, *IL-1*, *STAT3*, and *TNFAIP8*, indicating their impact on biological pathways associated with cell adhesion, movement, and signaling (83). The aberrant cytosine methylation of the IL - 6 promoter in RA resulted in the reduction of its transcription (89), while loss of cytosine methylation in the IL - 10 promoter correlated with the higher expression of IL - 10 (90). Several factors may impact the variation in the methylation pattern of these genes, which include weather fluctuations such as summer and winter conditions, physical activities, and staple diet. Vitamin D levels are known to have seasonal fluctuation, with the lowest in winter possibly due to reduced exposure to sunlight and UV, which can have important implications in bone metabolism and immunomodulatory effects on immune cells, which are all features of RA (91). Reduced vitamin D levels can be impacted by the epigenome or can impact the epigenome in multiple ways, as vitamin D signaling pathway genes have large CpG islands in their promoter regions, and the VDR genes or their protein can bidirectionally interact with chromatin modifiers or remodelers (92). Therefore, understanding the role of seasonal cycles in regulating the epigenome in the regulation of the expression of biological pathways associated with immune responses in driving RA pathology may be of tremendous interest.

Histone modifications are another important epigenetic mark that affects gene expression, resulting in variation in cellular phenotype. The epigenetic landscape of histones is modified through the mechanisms of acetylation, methylation, citrullination, phosphorylation, ubiquitinylation, and sumoylation, which can represent open and closed chromatin structure (92). The open chromatin structure makes chromatin accessible to transcription factors and can significantly increase gene expression. These include histone lysine residue acetylation (H3K9, H3K14, H4K5, and H4K16) and methylation (H2BK5, H3K4, H3K36, and H3K79), and phosphorylation of histone H3 threonine 3 (H3T3) and serine (H3S10 and H3S28), as well as histone 4 serine 1 (H4S1) and H2BK120 ubiquitinoylation. However, repressive histone marks correlate with heterochromatin state and gene repression, which include the methylation of H3K9, H3K27, and H4K20; the ubiquitination of H2AK119; and the sumoylation of H2AK126, H2BK6, and H2BK7 (93). The majority of the studies have focused on the acetylation and methylation of histones, while the citrullination of histones has also been reported in rheumatoid arthritis synovial fibroblasts (RASFs) (94). It is difficult to exactly demonstrate the RA-specific alterations of histone marks, as different types of histone modifications are colocalized throughout the genome in order to stabilize active or repressed chromatin states. To overcome this challenge, often, the expression of histone acetyltransferases (HATs) and histone deacetylases (HDACs) is studied. The activation of HDAC in RA patients has been reported (95). The decreased expression of HDAC1 and HDAC2 has been reported in RA synovial tissues, indicating a shift toward hyperacetylation (96). Active histone marks—including H4K4me3 and histone acetylation—are widely seen in the promoter region of transcription factor T-box transcription factor 5 (TBX5), and this TBX5 is also overexpressed in the synovial fibroblasts in RA (97). The overexpression of TBX5 can affect the expression of 790 genes, including *IL-8*, *CXCL12*, and *CCL20*, which play critical roles as inducers and regulators of chemokines, important in RA development. The presence of active histone marks (H3K4me3 and H3K27me3) in the promoter of *SFRP1* can increase the expression of SFRP1, leading to the inhibition of the Wnt signaling pathway in synovial fibroblasts in RA (98). Higher levels of active H3K4me3 marks in promoters of *MMP-1*, *MMP-3*, *MMP-9*, and *MMP-13*, along with reduced repressive modification H3K27me3 in promoters of *MMP-1* and *MMP-9*, have been reported in synovial fibroblasts in RA (99). Elevated levels of histone H4 acetylation have been associated with the upregulation of *MMP-1* in RA (100). These epigenetic signals in the genomic DNA are highly dynamic and show highly differential expression patterns in each cell type. Therefore, epigenetic profiling and unique mRNA expression patterns will be highly specific in an individual cell type according to various climatic conditions.

### MicroRNA in rheumatoid arthritis

MicroRNAs are short non-coding RNAs that typically regulate gene expression by either repressing the translation or causing the degradation of multiple target mRNAs. In rheumatic conditions, a set of miRNAs has been involved in the key regulation of downstream and upstream signaling pathways and in controlling chronic inflammation (101). MiRNA-24, miRNA-26a, and miRNA-125a-5p have been proposed as a potential diagnostic panel with sensitivity and specificity of 78.4% and 92.3%, respectively (102). MiRNA-21 and miRNA-146a determine the pro-inflammatory phenotype in RA by targeting Tregs (103, 104). MiRNA-146 and miRNA-155 are best described in synovial fibroblasts in RA. The increased expression of miRNA-203 (105), miRNA-221 (106), miRNA-663 (107), miRNA-222, and miRNA-323-3p (108) involved in immune processes has been reported in synovial fibroblasts in RA. The decreased expression of miRNA-124a, miRNA-34a^∗^, miRNA-152, miRNA-375, and miRNA-22 contributes to RA development (109). Studies have shown that serum miR-22 and miR-103a may predict RA development in susceptible individuals, while serum miR-16, miR-24, miR-125a, and miR-223 are indicators of early RA compared to established RA. In contrast, serum miR-223 levels are associated with disease activity and disease relapse. MiR-125b and miR-223 can act as drug response prediction markers for RA treatment. MiRNA-326 influences the increased expression of autoantibodies. Some of the miRNAs regulate other epigenetic events by targeting DNMTs, mediating DNA hypomethylation. MicroRNAs 29, 29b, and 143 are linked to the expression of DNMT3a and DNMT3b and indirectly linked with DNMT1 (110).

**Figure 3:**
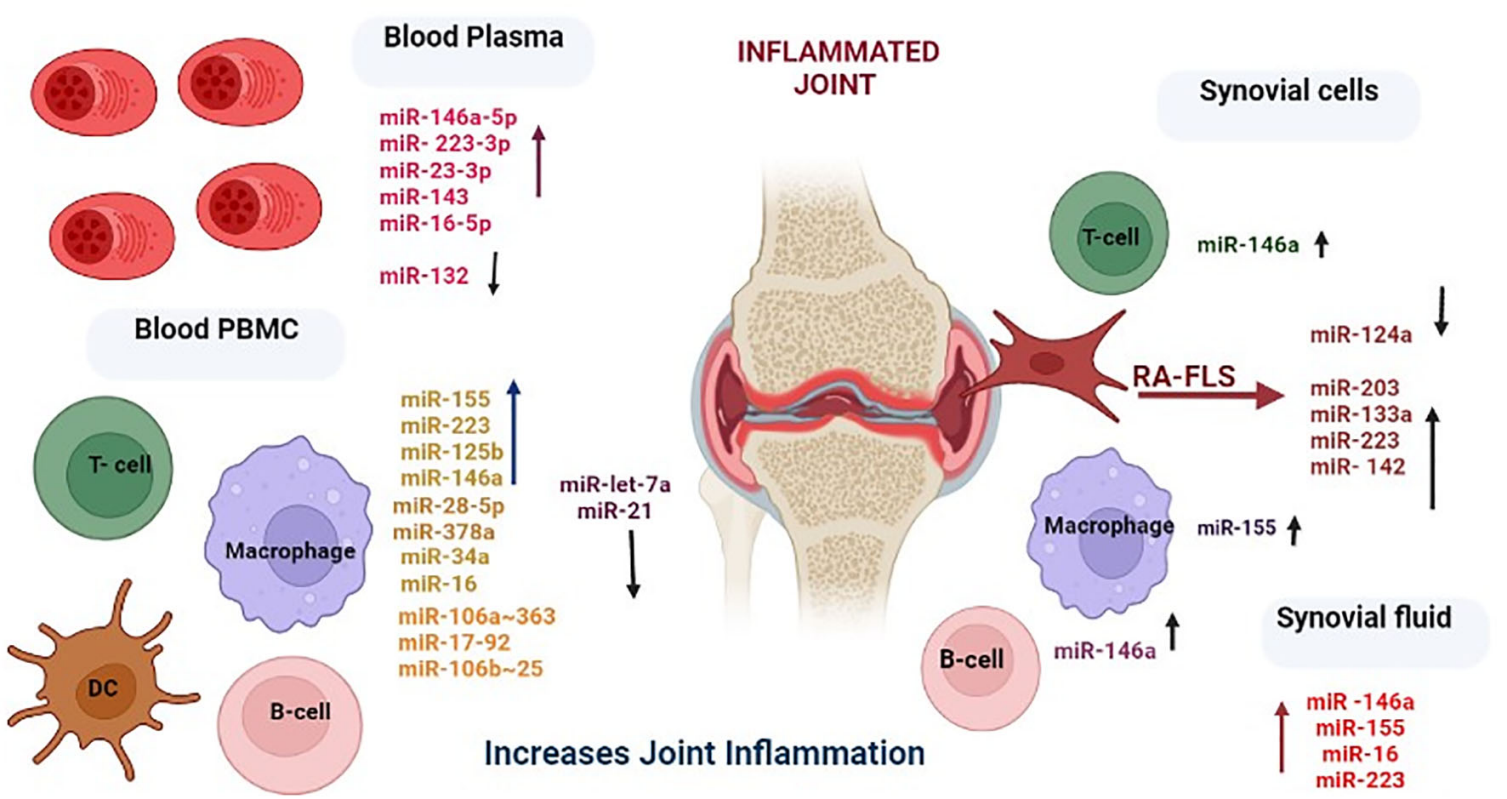
Differential expression levels of miRNAs and their corresponding cell types involved in rheumatoid arthritis. Created in BioRender. Priya, K. (2025) https://BioRender.com/l4bxpgr.

**Figure 4:**
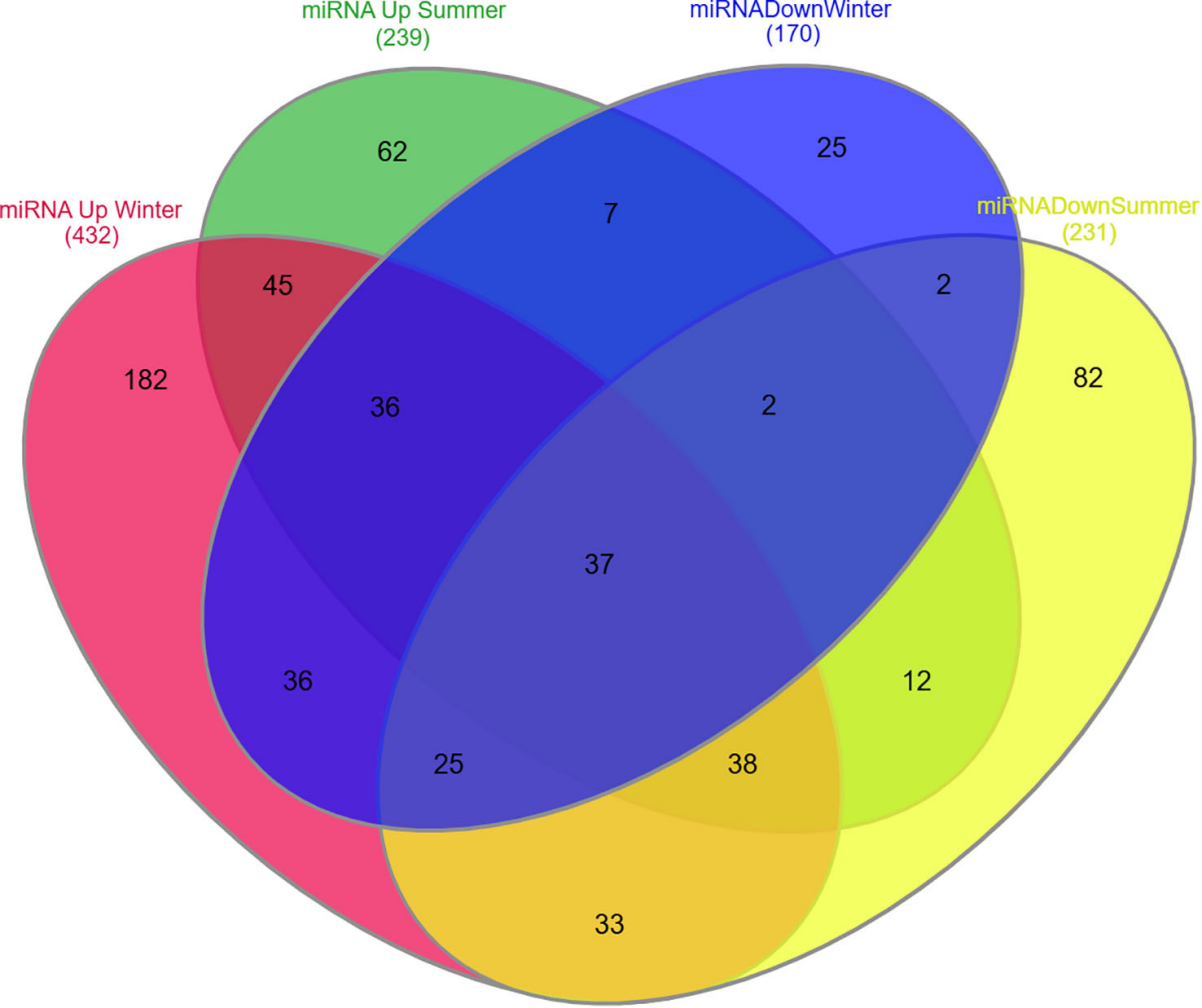
Venn diagram representing unique and common miRNA expression patterns during summer and winter seasons. Red: MiRNAs upregulated during winter season. Green: MiRNAs upregulated during summer season. Blue: MiRNAs downregulated during winter season. Yellow: MiRNAs downregulated during summer season.

The upregulated and downregulated miRNAs in the plasma, PBMCs, and synovial fluid of rheumatic arthritis patients are linked to various immune cells, as presented in **Figure 3**. A plethora of miRNA alterations that occur in cell types or tissues involved in RA pathogenesis are the consequence of disease progression. MiRNA changes may be sensitive indicators of the effects of acute and chronic environmental exposure. Therefore, miRNAs are valuable novel biomarkers for exposure. These miRNAs can also act as potential signatures and sensitive biomarkers for environmental factors like temperature, climatic variation, radiation, and day and length variations (111–118). It is possible that the reported miRNA may be influenced by seasonal fluctuations, as gene expressions are known to be influenced by seasons. To resolve this phenomenon, we considered all the genes that are differentially expressed during the summer and winter months and then verified how many of these genes are involved in RA pathogenesis and which miRNAs target these genes. Interestingly, some of these miRNAs have been demonstrated to be influenced by seasonal cycles (**Supplementary Table 2**) (107). The list of miRNAs that are uniquely and commonly influenced by seasonal variations is shown in **Figure 4** and **Supplementary Table 2**. The miRNAs and their target genes are extracted from the datalink (https://www.ccb.uni-saarland.de/mirtargetlink2). From this, we extrapolated that miRNAs can be differentially impacted by seasonal influences during the winter and summer seasons, which in turn can influence gene expression. These data show that miRNA expression is dependent on seasonal shift, reflecting its role in the disease pathogenesis of RA. This further adds to the evidence that seasonal cycles can disrupt the gene–environment interaction modulated by epigenetic drivers such as DNA methylation, histone modification, and miRNA on the genetically predisposed risk factors and thereby influence the seasonal impact on RA.

## Conclusion and future perspective

RA affects multiple joints with chronic inflammation and increased disease activity. Seasonal patterns can impact the phenotypes through genetic and epigenetic phenomena. Epigenetic phenomena rely heavily on the genetic makeup of an individual, which in turn will affect gene activity. In GWASs and candidate gene studies, many of the common variants have been found to be associated with RA, which will determine the genetic threshold of an individual. The risk variants in the genes upon the environmental trigger of seasons can trigger disease pathogenesis, increasing the burden of the disease during certain seasons. Therefore, understanding the contribution of genetic variants in genotype and phenotype plasticity in response to seasonality is necessary to understand the magnitude of the adaptive responses of an organism to climatic fluctuation. Unlike genetic changes, epigenetic modifications can also alter gene expression and lead to a spectrum of cellular events, which facilitate the adaptation of individual cells to their surroundings. Several lifestyle factors may modify epigenetic phenomena that impact transgenerational epigenetic inheritance. Therefore, understanding how these epigenetic modifiers communicate with each other and alter DNA architecture and gene expression is a key challenge in future treatment strategies in clinical settings for better outcomes of diseased individuals.

## References

[1] Gibofsky A. Epidemiology, pathophysiology, and diagnosis of rheumatoid arthritis: A Synopsis. Am J Manag Care. (2014) 20:S128–135., PMID: 25180621

[2] van Delft MA Huizinga TWJ. An overview of autoantibodies in rheumatoid arthritis. J Autoimmun. (2020) 110:102392. doi: 10.1016/j.jaut.2019.102392, PMID: 31911013

[3] Vossenaar ER Zendman AJW van Venrooij WJ. Citrullination, a possible functional link between susceptibility genes and rheumatoid arthritis. Arthritis Res Ther. (2004) 6:1–5. doi: 10.1186/ar1027, PMID: 14979924 PMC400418

[4] Choi R Lee SG Lee EH. Intraindividual changes in rheumatoid factor and anti-cyclic citrullinated peptide antibody tests in Korean patients visiting local clinics and hospitals. J Clin Med. (2022) 11:832. doi: 10.3390/jcm11030832, PMID: 35160283 PMC8836817

[5] Arend WP Firestein GS. Pre-rheumatoid arthritis: predisposition and transition to clinical synovitis. Nat Rev Rheumatol. (2012) 8:573–86. doi: 10.1038/nrrheum.2012.34, PMID: 22907289

[6] Lawrence JS. Heberden Oration. Rheumatoid arthritis–nature or nurture? Ann Rheum Dis. (1970) 29:357–79. doi: 10.1136/ard.29.4.357, PMID: 4916766 PMC1031320

[7] MacGregor AJ Snieder H Rigby AS Koskenvuo M Kaprio J Aho K. Characterizing the quantitative genetic contribution to rheumatoid arthritis using data from twins. Arthritis Rheum. (2000) 43:30–7. doi: 10.1002/1529-0131(200001)43:1, PMID: 10643697

[8] Hawley DJ Wolfe F F Lue FA Moldofsky H. Seasonal symptom severity in patients with rheumatic diseases: a study of 1,424 patients. J Rheumatol. (2001) 28:1900–9., PMID: 11508598

[9] Alegría-Torres JA Baccarelli A Bollati V. Epigenetics and lifestyle. Epigenomics. (2011) 3:267–77. doi: 10.2217/epi.11.22, PMID: 22122337 PMC3752894

[10] Weyand CM Goronzy JJ. The immunology of rheumatoid arthritis. Nat Immunol. (2021) 22:10–8. doi: 10.1038/s41590-020-00816-x, PMID: 33257900 PMC8557973

[11] GBD 2021 Forecasting Collaborators. Burden of disease scenarios for 204 countries and territories, 2022 - 2050: a forecasting analysis for the Global Burden of Disease Study 2021. Lancet. (2024) 403:2204–56. doi: 10.1016/S0140-6736(24)00685-8, PMID: 38762325 PMC11121021

[12] Shi W Liang X Zhang H Li H. Burden of rheumatoid arthritis in India from 1990 to 2021: insights from the Global Burden of Disease Database. Front Med. (2025) 12:1526218. doi: 10.3389/fmed.2025.1526218, PMID: 40027898 PMC11868046

[13] Riise T Jacobsen BK Gran JT. Incidence and prevalence of rheumatoid arthritis in the county of Troms, Northern Norway. J Rheumatol. (2000). 27(6):1386-9, PMID: 10852258

[14] Symmons DP Turner G Webb R Asten P Barrett E Lunt M. The prevalence of rheumatoid arthritis in the United Kingdom: new estimates for a new century. Rheumatol. (2002) 41:793–800. doi: 10.1093/rheumatology/41.7.793, PMID: 12096230

[15] Andrianakos A Trontzas P Christogiannis F Dantis P Voudouris C Georgountzos A. Prevalence of rheumatic diseases in Greece: a cross-sectional population based epidemiological study. The ESORDIG Study. J.Rheumatol. (2003) 30:1589–601. 12858464

[16] Shichikawa K Inoue K Hirota S Maeda A Ota H Kimura M. Changes in the incidence and prevalence of rheumatoid arthritis in Kamitonda, Wakayama, Japan, 1965 - 1996. Ann Rheum Dis. (1999) 58:751–6. doi: 10.1136/ard.58.12.751, PMID: 10577961 PMC1752814

[17] Alamanos Y Voulgari PV Drosos AA. Incidence and prevalence of rheumatoid arthritis, based on the 1987 American College of Rheumatology criteria: a systematic review. Semin Arthritis Rheumatol. (2006) 36:182–8. doi: 10.1016/j.semarthrit.2006.08.006, PMID: 17045630

[18] Costenbader KH Chang SC Laden F Puett R Karlson EW. Geographic variation in rheumatoid arthritis incidence among women in the United States. Arch Intern Med. (2008) 168:1664–70. doi: 10.1001/archinte.168.15.1664, PMID: 18695080 PMC2732358

[19] Patberg WR Rasker JJ. Weather effects in rheumatoid arthritis: from controversy to consensus. A review. J Rheumatol. (2004) 31:1327–34., PMID: 15229951

[20] Venetsanopoulou AI Alamanos Y Voulgari PV Drosos AA. Epidemiology and risk factors for rheumatoid arthritis development. Mediterr J Rheumatol. (.(2023) 34:404–13. doi: 10.31138/mjr.301223.eaf, PMID: 38282942 PMC10815538

[21] Gwinnutt JM Wieczorek M Rodríguez-Carrio J Balanescu A Bischoff-Ferrari HA Boonen A. Effects of diet on the outcomes of rheumatic and musculoskeletal diseases (RMDs): systematic review and meta-analyses informing the 2021 EULAR recommend dations for lifestyle improvements in people with RMDs. . RMD Open. (2022) 8:e002167. doi: 10.1136/rmdopen-2021-002167, PMID: 35654458 PMC9096533

[22] Hewagama A Richardson B. The genetics and epigenetics of autoimmune diseases. J Autoimmun. (2009) 33:3–11. doi: 10.1016/j.jaut.2009.03.007, PMID: 19349147 PMC2819418

[23] Weyand CM Goronzy JJ. Association of MHC and rheumatoid arthritis: HLA polymor- phisms in phenotypic variants of rheumatoid arthritis. Arthritis Res Ther. (2000) 2:212–216. doi: 10.1186/ar90, PMID: 11094432 PMC130005

[24] Wysocki T Olesińska M Paradowska-Gorycka A. Current understanding of an emerging role of HLA-DRB1 gene in rheumatoid arthritis–from research to clinical practice. Cells. (2020) 9:1127. doi: 10.3390/cells9051127, PMID: 32370106 PMC7291248

[25] Gregersen PK Silver J Winchester RJ. The shared epitope hypothesis. An approach to understanding the molecular genetics of susceptibility to rheumatoid arthritis. Arthritis Rheumatol. (1987) 30:1205–13. doi: 10.1002/art.1780301102, PMID: 2446635

[26] Wucherpfennig KW Strominger JL. Selective binding of self- peptides to disease-associated major histocompatibility complex (MHC) molecules: a mechanism for MHC-linked susceptibility to human autoimmune diseases. J Exp Med. (1995) 181:1597 – 601. doi: 10.1084/jem.181.5.1597, PMID: 7722439 PMC2191999

[27] Wagner UG Koetz K Weyand CM Goronzy JJ. Perturbation of the T cell repertoire in rheumatoid arthritis. Proc Natl Acad Sci USA. (1998) 95:14447–52. doi: 10.1073/pnas, PMID: 9826720 PMC24393

[28] Holoshitz J. The rheumatoid arthritis HLA-DRB1 shared epitope. Curr Opin Rheumatol. (2010) 22:293–98. doi: 10.1097/BOR.0b013e328336ba63, PMID: 20061955 PMC2921962

[29] Reinsmoen NL Bach FH. Structural model for T-cell recognition of HLA class II associated alloepitopes. Hum Immunol. (1990) 27:51–72. doi: 10.1016/0198-8859(90)90095-7, PMID: 1689285

[30] Stahl EA Raychaudhuri S Remmers EF Xie G Eyre S Thomson BP. Genome-wide association study meta-analysis identifies seven new rheumatoid arthritis risk loci. Nat Genet. (2010) 42:508–14. doi: 10.1038/ng.582, PMID: 20453842 PMC4243840

[31] Reviron D Foutrier C Guis S Mercier P Roudier J. DRB1 alleles in polymyalgia rheumatica and rheumatoid arthritis in southern France. Eur J Immunogen. (2001) 28:83–87. doi: 10.1046/j.1365-2370.2001.00228.x, PMID: 11251689

[32] Delgado-Vega AM Anaya JM. Meta-analysis of HLA-DRB1 polymorphism in Latin American patients with rheumatoid arthritis. Autoimmun Rev. (2007) 6:402–8. doi: 10.1016/j.autrev.2006.11.004, PMID: 17537386

[33] Jun KR Choi SE Cha CH Oh HB Heo YS Ahn HY. Meta-analysis of the association between HLA-DRB1 allele and rheumatoid arthritis susceptibility in Asian populations. J Korean Med Sci. (2007) 22:973–80. doi: 10.3346/jkms.2007.22.6.973, PMID: 18162709 PMC2694263

[34] Cruz GI Shao X Quach H Ho KA Sterba K Noble JA. Increased risk of rheumatoid arthritis among mothers with children who carry DRB1 risk-associated alleles. Ann Rheum Dis. (2017) 76:1405–10. doi: 10.1136/annrheumdis-2016-210662, PMID: 28391248 PMC7450788

[35] Lee HS Lee KW Song GG Kim HA Kim SY Bae SC. Increased susceptibility to rheumatoid arthritis in Koreans heterozygous for HLA–DRB1* 0405 and *0901. Arthritis Rheumatol. (2004) 50:3468–75. doi: 10.1002/art.20608, PMID: 15529363

[36] Kim K Bang SY Yoo DH Cho SK Choi CB Sung YK. Imputing variants in HLA-DR beta genes reveals that HLA-DRB1 is solely associated with rheumatoid arthritis and systemic lupus erythematosus. PloS One. (2016) 11:e0150283. doi: 10.1371/journal, PMID: 26919467 PMC4769216

[37] Hong GH Park MH Takeuchi F Oh MD Song YW Nabeta H. Association of specific amino acid sequence of HLA-DR with rheumatoid arthritis in Koreans and its diagnostic value. J Rheumatol. (1996) 23:1699–703., PMID: 8895143

[38] Kim HY Kim TG Park SH Lee SH Cho CS Han H. Predominance of HLA-DRB1*0405 in Korean patients with rheumatoid arthritis. Ann Rheum Dis. (1995) 54:988–90. doi: 10.1136/ard.54.12.988, PMID: 8546532 PMC1010065

[39] Chan SH Lin YN Wee GB Koh WH Boey ML. HLA class 2 genes in Singaporean Chinese rheumatoid arthritis. Br J Rheumatol. (1994) 33:713–7. doi: 10.1093/rheumatology/33.8.713, PMID: 8055196

[40] de Vries N Renningen KS Tilanus MG Bouwens-Rambouts A Segal R Egeland T. HLA-DR1 and rheumatoid arthntis in Israeli Jews: Sequencing reveals that DRB1* 0102 is the predominant HLA-DR1 subtype. Tissue Antigens. (1993) 41:26–30. doi: 10.1111/j.1399-0039.1993.tb01973.x, PMID: 8456440

[41] Ferucci ED Templin DW Lanier AP. Rheumatoid arthritis in American Indians and Alaska Natives: a review of the literature. Sem Arth Rheumatol. (2005) 34:662 – 67. doi: 10.1016/j.semarthrit.2004.08.003, PMID: 15692959

[42] Nelson JL Boyer G Templin D Lanier A Barrington R Nisperos B. HLA antigens in Tlingit Indians with rheumatoid arthritis. Tissue Antigens. (1992) 40:57–63. doi: 10.1111/j.1399-0039.1992.tb01960.x, PMID: 1412417

[43] Hughes LB Morrison D Kelley JM Padilla MA Vaughan LK Westfall AO. The HLA–DRB1 shared epitope is associated with susceptibility to rheumatoid arthritis in African Americans through European genetic admixture. Arthritis Rheumatol. (2008) 58:349–358. doi: 10.1002/art.23166, PMID: 18240241 PMC3726059

[44] Singwe-Ngandeu M Finckh A Bas S Tiercy JM Gabay C. Diagnostic value of anti-cyclic citrullinated peptides and association with HLA-DRB1 shared epitope alleles in African rheumatoid arthritis patients. Arthritis Res Ther. (2010) 12:R36. doi: 10.1186/ar2945, PMID: 20196860 PMC2888183

[45] Higami K Hakoda M Matsuda Y Ueda H Kashiwazaki S. Lack of association of HLA DRB1 genotype with radiologic progression in Japanese patients with early rheumatoid arthritis. Arthritis Rheumatol. (1997) 40:2241–2247. doi: 10.1002/art.1780401220, PMID: 9416863

[46] Huizinga TW Amos CI van der Helm-van Mil AH Chen W van Gaalen FA Jawaheer D. Refining the complex rheumatoid arthritis phenotype based on specificity of the HLA-DRB1 shared epitope for antibodies to citrullinated proteins. Arthritis Rheumatol. (2005) 52:3433–38. doi: 10.1002/art.21385, PMID: 16255021

[47] Wipfler K Pedro S Sbarigia U Zazzetti F Sheahan A Lin I. Pos0477 relationship between shared epitope status and refractory rheumatoid arthritis. Ann Rheumatic Dis. (2023) 82:499. doi: 10.1136/annrheumdis-2023-eular.4095

[48] Oka S Furukawa H Kawasaki A Shimada K Sugii S Hashimoto A. Protective effect of the HLA-DRB1* 13: 02 allele in Japanese rheumatoid arthritis patients. PloS One. (2014) 9:e99453. doi: 10.1371/journal.pone.0099453, PMID: 24911054 PMC4049831

[49] van der Woude D Lie BA Lundström E Balsa A Feitsma AL Houwing-Duistermaat JJ. Protection against anti–citrullinated protein antibody–positive rheumatoid arthritis is predominantly associated with HLA–DRB1*1301: A meta-analysis of HLA–DRB1 associations with anti–citrullinated protein antibody–positive and anti–citrullinated protein antibody–negative rheumatoid arthritis in four European populations. Arthritis Rheumatol. (2010) 62:1236–45. doi: 10.1002/art.27366, PMID: 20131291

[50] Cano-Gamez E Trynka G. From GWAS to function: using functional genomics to identify the mechanisms underlying complex diseases. Front Gen. (2020) 11:424. doi: 10.3389/fgene.2020.00424, PMID: 32477401 PMC7237642

[51] Eichler EE Flint J Gibson G Kong A Leal SM Moore JH. Missing heritability and strategies for finding the underlying causes of complex disease. Nat Rev Genet. (2010) 11:446–50. doi: 10.1038/nrg2809, PMID: 20479774 PMC2942068

[52] Hirschhorn JN. Genomewide association studies–illuminating biologic pathways. . N Engl J Med. (2009) 360:1699–701. doi: 10.1056/NEJMp0808934, PMID: 19369661

[53] Katsumoto TR Stolyar L Dandeniya CL Wong HN Lanata CM Falasinnu T. Impact of climate change on rheumatic diseases: A scoping review. J Climate Change Health. (2024) 19:100338. doi: 10.1016/j.joclim.2024.100338 PMC12851271 41647313

[54] Morimoto HA. probabilistic approach for links between rheumatic diseases and weather. Int Med Care. (2017) 1:1–4. doi: 10.15761/IMC.1000104

[55] Takahashi K Kubo T Arai Y Kitajima I Takigawa M Imanishi J. Hydrostatic pressure induces expression of interleukin 6 and tumour necrosis factor alpha mRNAs in a chondrocyte-like cell line. Ann Rheum Dis. (1988) 57:231–6. doi: 10.1136/ard.57.4.231, PMID: 9709180 PMC1752582

[56] Yamamoto T Kita M Kimura I Oseko F Terauchi R Takahashi K. Mechanical stress induces expression of cytokines in human periodontal ligament cells. Oral Dis. (2006) 12:171–175. doi: 10.1111/j.1601-0825.2005.01179.x, PMID: 16476039

[57] Frangogiannisa NG Smithb CW Entman ML. The inflammatory response in myocardial infarction. Cardiovasc Res. (2002) 53:31–47. doi: 10.1016/s0008-6363(01)00434-5, PMID: 11744011

[58] Iikuni N Kakajima A Inoue E Tanaka E Okamoto H Hara M. What’s in season for rheumatoid arthritis patients? Seasonal fluctuation in disease activity. J Rheumatol. (2007) 46:846–8. doi: 10.1093/rheumatology/kel414, PMID: 17264092

[59] Mouterde G Lukas C Logeart I Flipo RM Rincheval N Daures JP. Predictors of radiographic progression in the ESPOIR cohort: the season of first symptoms may influence the short-term outcome in early arthritis. Ann Rheum Dis. (2011) 70:1251–56. doi: 10.1136/ard.2010.144402, PMID: 21515603

[60] Azzouzi H Ichchou L. Seasonal and weather effects on rheumatoid arthritis: myth or reality? Pain Res Manage. (2020) 2020:5763080. doi: 10.1155/2020/5763080, PMID: 32963656 PMC7492902

[61] Dopico XC Evangelou M Ferreira RC Guo H Pekalski ML Smyth DJ. Widespread seasonal gene expression reveals annual differences in human immunity and physiology. Nat Commun. (2015) 12;6:7000. doi: 10.1038/ncomms8000, PMID: 25965853 PMC4432600

[62] Watad A Azrielant S Bragazzi NL Sharif K David P Katz I. Seasonality autoimmune diseases: contribution four seasons to mosaic autoimmunity. J Autoimmun. (2017) 82:13–30. doi: 10.1016/j.jaut.2017.06.001, PMID: 28624334

[63] Brincas HM Augusto DG Mathias C Cavalli IJ Lima RS Kuroda F. Genetic variant in microRNA-146a is associated with sporadic breast cancer in a southern Brazilian population. Genet.Mol.Biol. (2020) 42:e20190278. doi: 10.1590/1678-4685-GMB-2019-0278, PMID: 32142098 PMC7198002

[64] Ter Horst R Jaeger M Smeekens SP Oosting M Swertz MA Li Y. Host and environmental factors influencing individual human cytokine responses. Cell. (2016) 167:1111–24. doi: 10.1016/j.cell.2016.10.018, PMID: 27814508 PMC5787854

[65] Krishna Priya EK Srinivas L Rajesh S Sasikala K Banerjee M. Pro-inflammatory cytokine response pre-dominates immuno-genetic pathway in development of rheumatoid arthritis. Mol Bio Rep. (2020) 47:8669–77. doi: 10.1007/s11033-020-05909-2, PMID: 33074413

[66] Vatsiou AI Bazin E Gaggiotti OE. Changes in selective pressures associated with human population expansion may explain metabolic and immune related pathways enriched for signatures of positive selection. BMC Genomics. (2016) 17:504. doi: 10.1186/s12864-016-2783-2, PMID: 27444955 PMC4955149

[67] Kawali A Zulaikha V Srinivasan S Mahendradas P Kumar J Shetty R. HLA-B27-related uveitis and seasonal variation–an Indian perspective. Indian J Ophthalmol. (2020) 68:1863–1866. doi: 10.4103/ijo.IJO_388_20, PMID: 32823403 PMC7690497

[68] Ingolotti M Ormaechea MS Fernandez JP Portela M Lupinacci A Couto CA. Clinical features and seasonal variation of HLA B27 positive associated anterior uveitis in an Argentine Tertiary University Hospital. Invest.Ophthalmol.Vis.Sci. (2016) 57:3292.

[69] Gonzalez-Gay MA Garcia-Porrua C Hajeer AH. Influence of human leukocyte antigen-DRB1 on the susceptibility and severity of rheumatoid arthritis. Semin Arthritis Rheumatol. (2002) 31:355–60. doi: 10.1053/sarh.2002.32552, PMID: 12077707

[70] Plant MJ Jones PW Saklatvala J Ollier WE Dawes PT. Patterns of radiological progression in early rheumatoid arthritis: results of an 8 years prospective study. J Rheumatol. (1998) 25:417–26., PMID: 9517757

[71] de Vries RR Huizinga TW Toes RE. Redefining the HLA and RA association: to be or not to be anti-CCP positive. J Autoimmun. (2005) 25 Suppl:21–5. doi: 10.1016/j.jaut.2005.09.005, PMID: 16257178

[72] Snir O Widhe M von Spee C Lindberg J Padyukov L Lundberg K. Multiple antibody reactivities to citrullinated antigens in sera from patients with rheumatoid arthritis: association with HLA-DRB1 alleles. Ann Rheum Dis. (2009) 68:736–43. doi: 10.1136/ard.2008.091355, PMID: 18635594

[73] Weinberg CR Dornan TL Hansen JA Raghu PK Palmer JP. HLA-related heterogeneity in seasonal patterns of diagnosis in Type 1 (insulin-dependent) diabetes. Diabetologia. (1984) 26:199–202. doi: 10.1007/BF00252407, PMID: 6609096

[74] Viatte S Plant D Raychaudhuri S. Genetics and epigenetics of rheumatoid arthritis. Nat Rev Rheumatol. (2013) 9:141–53. doi: 10.1038/nrrheum.2012.237, PMID: 23381558 PMC3694322

[75] Bell JT Spector TD. A twin approach to unravelling epigenetics. Trends Genet. (2011) 27:116–25. doi: 10.1016/j.tig.2010.12.005, PMID: 21257220 PMC3063335

[76] Gillette TG Hill JA. Readers, writers, and erasers: chromatin as the whiteboard of heart disease. Circ Res. (2015) 116:1245–53. doi: 10.1161/CIRCRESAHA.116.303630, PMID: 25814685 PMC4380191

[77] Glant TT Mikecz K Rauch TA. Epigenetics in the pathogenesis of rheumatoid arthritis. BMC Med. (2014) 12:35. doi: 10.1186/1741-7015-12-35, PMID: 24568138 PMC3936819

[78] Karlson EW Deane K. Environmental and gene-environment interactions and risk of rheumatoid arthritis. Rheum Dis Clin North Am. (2012) 38:405–26. doi: 10.1016/j.rdc.2012.04.002, PMID: 22819092 PMC3402910

[79] Banerjee M. Genetics of epigenome might hold the clue for determining the threshold of environmental impact. Epigenomics. (2019) 11:983–6. doi: 10.2217/epi-2019-0123, PMID: 31282764

[80] Ospelt C. Epigenetic biomarkers in rheumatology-the future? Swiss Med Wkly. (2016) 1:146. doi: 10.4414/smw.2016.14312, PMID: 27249518

[81] Moore L Le T Fan G. DNA methylation and its basic function. Neuropsycho-pharmacol. (2013) 38:23–38. doi: 10.1038/npp.2012.112, PMID: 22781841 PMC3521964

[82] de Andres MC Perez-Pampin E Calaza M Santaclara FJ Ortea I Gomez-Reino JJ. Assessment of global DNA methylation in peripheral blood cell subpopulations of early rheumatoid arthritis before and after methotrexate. Arthritis Res Ther. (2015) 17:233. doi: 10.1186/s13075-015-0748-5, PMID: 26330155 PMC4556005

[83] Glossop JR Emes RD Nixon NB Packham JC Fryer AA Mattey DL. Genome-wide profiling in treatment-naive early rheumatoid arthritis reveals DNA methylome changes in T and B lymphocytes. Epigenomics. (2016) 8:209–24. doi: 10.2217/epi.15.103, PMID: 26556652

[84] Cribbs AP Kennedy A Penn H Read JE Amjadi P Green P. Treg cell function in rheumatoid arthritis is compromised by CTLA - 4 promoter methylation resulting in a failure to activate the indoleamine 2,3-dioxygenase pathway. Arthritis Rheumatol. (2014) 66:2344–54. doi: 10.1002/art.38715, PMID: 24891289

[85] Cribbs AP Kennedy A Penn H Amjadi P Green P Read JE. Methotrexate restores regulatory T cell function through demethylation of the FoxP3 upstream enhancer in patients with rheumatoid arthritis. Arthritis Rheumatol. (2015) 67:1182–1192. doi: 10.1002/art.39031, PMID: 25604080

[86] Raychaudhuri S Sandor C Stahl EA Freudenberg J Lee HS Jia X. Five amino acids in three HLA proteins explain most of the association between MHC and seropositive rheumatoid arthritis. Nat Genet. (2012) 44:291–6. doi: 10.1038/ng.1076, PMID: 22286218 PMC3288335

[87] van Steenbergen HW Luijk R Shoemaker R Heijmans BT Huizinga TWJ van der Helm-van Mil AHM. Differential methylation within the major histocompatibility complex region in rheumatoid arthritis: a replication study. Rheumatology. (2014) 53:2317–8. doi: 10.1093/rheumatology/keu380, PMID: 25273994

[88] Baron U Türbachova I Hellwag A Eckhardt F Berlin K Hoffmuller U. DNA methylation analysis as a tool for cell typing. Epigenetics. (2006) 1:56–61. doi: 10.4161/epi.1.1.2643, PMID: 17998806

[89] Nile CJ Read RC Akil M Duff GW Wilson AG. Methylation status of a single CpG site in the IL6 promoter is related to IL6 messenger RNA levels and rheumatoid arthritis. Arthritis Rheumatol. (2008) 58:2686–93. doi: 10.1002/art.23758, PMID: 18759290

[90] Chen H Fu L Li S Ma C Zhang J Cong B. Hypomethylation of proximal CpG motif of interleukin-10 promoter regulates its expression in human rheumatoid arthritis. Acta Pharmacol Sin. (2011) 32:1373–80. doi: 10.1038/aps.2011.98, PMID: 21986577 PMC4002740

[91] Cutolo M Soldano S Sulli A Smith V Gotelli E. Influence of seasonal vitamin D changes on clinical manifestations of rheumatoid arthritis and systemic sclerosis. Front Immunol. (2021) 12:683665. doi: 10.3389/fimmu.2021.683665, PMID: 34267753 PMC8276051

[92] Fetahu IS Höbaus J Kállay E. Vitamin D and the epigenome. Front Physiol. (2014) 5:164. doi: 10.3389/fphys.2014.00164, PMID: 24808866 PMC4010791

[93] Karouzakis E Trenkmann M Gay RE Michel BA Gay S Neidhart M. Epigenome analysis reveals TBX5 as a novel transcription factor involved in the activation of rheumatoid arthritis synovial fibroblasts. J Immunol. (2014) 193:4945–51. doi: 10.4049/jimmunol.1400066, PMID: 25320281

[94] Araki Y Mimura T. The mechanisms underlying chronic inflammation in rheumatoid arthritis from the perspective of the epigenetic landscape. J.Immunol.Res. (2016) 2016:6290682. doi: 10.1155/2016/6290682, PMID: 28116320 PMC5225373

[95] Wang F Chen FF Gao WB Wang HY Zhao NW Xu M. Identification of citrulli- nated peptides in the synovial fluid of patients with rheumatoid arthritis using LC-MALDI- TOF/TOF. Clin Rheumatol. (2016) 35:2185–94. doi: 10.1007/s10067-016-3247-4, PMID: 27060082 PMC4989008

[96] Toussirot E Abbas W Khan KA Tissot M Jeudy A Baud L. Imbalance between HAT and HDAC activities in the PBMCs of patients with ankylosing spondylitis or rheumatoid arthritis and influence of HDAC inhibitors on TNF alpha production. PloS One. (2013) 8:e70939. doi: 10.1371/journal.pone.0070939, PMID: 24039666 PMC3748901

[97] Kawabata T Nishida K Takasugi K Ogawa H Sada K Kadota Y. Increased activity, and expression of histone deacetylase1 in relation to tumor necrosis factor-alpha in synovial tissue of rheumatoid arthritis. Arthritis Res Ther. (2010) 12:R133. doi: 10.1186/ar3071, PMID: 20609223 PMC2945023

[98] Karouzakis E Trenkmann M Gay RE Michel BA Gay S Neidhart M. Epigenome analysis reveals TBX5 as a novel transcription factor involved in the activation of rheumatoid arthritis synovial fibroblasts. J Immunol. (2014) 193:4945–1951. doi: 10.4049/jimmunol.1400066, PMID: 25320281

[99] Trenkmann M Brock M Gay RE Kolling C Speich R Michel BA. Expression and function of EZH2 in synovial fibroblasts: epigenetic repression of the Wnt inhibitor SFRP1 in rheumatoid arthritis. Ann Rheumatol Dis. (2011) 70:1482–88. doi: 10.1136/ard.2010, PMID: 21515604

[100] Araki Y Wada TT Aizaki Y Sato K Yokota K Fujimoto K. Histone methylation and STAT3 differentially regulate IL - 6-induced MMP gene activation in rheumatoid arthritis synovial fibroblasts. Arthritis Rheumatol. (2016) 68:1111–23. doi: 10.1002/art, PMID: 26713842

[101] Maciejewska-Rodrigues H Karouzakis E Strietholt S Hemmatazad H Neidhart M Ospelt C. Epigenetics and rheumatoid arthritis: the role of SENP1 in the regulation of MMP - 1 expression. J Autoimmun. (2010) 35:15–22. doi: 10.1016/j.jaut.2009.12.010, PMID: 20079608

[102] Cunningham CC Wade S Floudas A Orr C McGarry T Wade S. Serum miRNA signature in Rheumatoid Arthritis and “At -Risk Individuals. .Front.Immunol. (2021) 12:633201. doi: 10.3389/fimmu.2021.633201, PMID: 33746971 PMC7966707

[103] Murata K Furu M Yoshitomi H Ishikawa M Shibuya H Hashimoto M. Comprehensive microRNA analysis identifies miR-24 and miR-125a-5p as plasma biomarkers for rheumatoid arthritis. PloS One. (2013) 8:e69118. doi: 10.1371/journal, PMID: 23874885 PMC3715465

[104] Salehi E Eftekhari R Oraei M Gharib A Bidad K. MicroRNAs in rheumatoid arthritis. Clin Rheumatol. (2015) 34:615–28. doi: 10.1007/s10067-015-2898-x, PMID: 25736037

[105] Zhou Q Haupt S Kreuzer JT Hammitzsch A Proft F Neumann C. Decreased expression of miR-146a and miR-155 contributes to an abnormal Treg phenotype in patients with rheumatoid arthritis. Ann Rheumatol Dis. (2015) 74:1265–74. doi: 10.1136/annrheumdis-2013-204377, PMID: 24562503

[106] Stanczyk J Ospelt C Karouzakis E Filer A Raza K Kolling C. Altered expression of microRNA-203 in rheumatoid arthritis synovial fibroblasts and its role in fibroblast activation. Arthritis Rheumatol. (2011) 63:373–81. doi: 10.1002/art.30115, PMID: 21279994 PMC3116142

[107] Yang S Yang Y. Downregulation of microRNA-221 decreases migration and invasion in fibroblast-like synoviocytes in rheumatoid arthritis. Mol.Med.Rep. (2015) 12:2395 – 401. doi: 10.3892/mmr.2015.3642, PMID: 25891943

[108] Miao C Shi W Xiong Y Yu H Zhang X Qin M. MicroRNA-663 activates the canonical Wnt signaling through the adenomatous polyposis coli suppression. Immunol.Lett. (2015) 166:45–54. doi: 10.1016/j.imlet.2015.05.011, PMID: 26028359

[109] Pandis I Ospelt C Karagianni N Denis MC Reczko M Camps C. Identification of microRNA-221/222 and microRNA-323-3p association with rheumatoid arthritis via predictions using the human tumour necrosis factor transgenic mouse model. Ann Rheumatol Dis. (2012) 71:1716–23. doi: 10.1136/annrheumdis-2011-200803, PMID: 22562984

[110] Garzon R Liu S Fabbri M Liu Z Heaphy CEA Callegari E. MicroRNA-29b induces global DNA hypomethylation and tumor suppressor gene reexpression in acute myeloid leukaemia by targeting directly DNMT3A and 3B and indirectly DNMT1. Blood. (2009) 1139:6411–8. doi: 10.1182/blood-2008-07-170589, PMID: 19211935 PMC2710934

[111] Ng EK Tsang WP Ng SS Jin HC Yu J Li JJ. MicroRNA-143 targets DNA methyltransferases 3A in colorectal cancer. Br J Cancer. (2009) 101:699–706. doi: 10.1038/sj.bjc.6605195, PMID: 19638978 PMC2736825

[112] Vrijens K Bollati V Nawrot TS. MicroRNAs as potential signatures of environmental exposure or effect: a systematic review. Environ Health Perspect. (2015) 123:399–411. doi: 10.1289/ehp.1408459, PMID: 25616258 PMC4421768

[113] Jia M Wang Z. MicroRNAs as biomarkers for ionizing radiation injury. . Front Cell Dev Biol. (2022) 10:861451. doi: 10.3389/fcell.2022.861451, PMID: 35309926 PMC8927810

[114] Miguel V Cui JY Daimiel L Espinosa-Díez C Fernández-Hernando C Kavanagh TJ. The role of microRNAs in environmental risk factors, noise-induced hearing loss, and mental stress. Antioxid Redox Signal. (2018) 28:773–96. doi: 10.1089/ars.2017.7175, PMID: 28562070 PMC5911706

[115] Letelier P Saldías R Loren P Riquelme I Guzmán N. MicroRNAs as potential biomarker of environmental exposure to polycyclic aromatic hydrocarbons and their link with inflammation and lung cancer. Int.J.Mol.Sci. (2023) 24:16984. doi: 10.3390/ijms242316984, PMID: 38069307 PMC10707120

[116] Ludwig N Hecksteden A Kahraman M Fehlmann T Laufer T Kern F. Spring is in the air: seasonal profiles indicate vernal change of miRNA activity. RNA Biol. (2019) 16:1034–43. doi: 10.1080/15476286.2019.1612217, PMID: 31035857 PMC6602417

[117] Vicente R Noël D Pers YM Apparailly F Jorgensen C. Deregulation and therapeutic potential of microRNAs in arthritic diseases. Nat.Rev.Rheumatol. (2016) 12:211–20. doi: 10.1038/nrrheum.2016.119, PMID: 26698025

[118] Fabbri M Garzon R Cimmino A Liu Z Zanesi N Callegari E. MicroRNA-29 family reverts aberrant methylation in lung cancer by targeting DNA methyltransferases 3A and 3B. Proc Natl Acad Sci USA. (2007) 104:15805–810. doi: 10.1073/pnas.0707628104, PMID: 17890317 PMC2000384

